# Asymptomatic COVID-19 Patients Can Contaminate Their Surroundings: an Environment Sampling Study

**DOI:** 10.1128/mSphere.00442-20

**Published:** 2020-06-24

**Authors:** Li Wei, Ji Lin, Xiaofei Duan, Wenzhi Huang, Xiaojun Lu, Juan Zhou, Zhiyong Zong

**Affiliations:** a Department of Infection Control, West China Hospital, Sichuan University, Chengdu, China; b Department of Infection Control, Chengdu Public Health Clinical Medical Center, Chengdu, China; c Department of Laboratory Medicine, West China Hospital, Sichuan University, Chengdu, China; d Center of Infectious Diseases, West China Hospital, Sichuan University, Chengdu, China; e Center for Pathogen Research, West China Hospital, Sichuan University, Chengdu, China; University of Wisconsin—Madison

**Keywords:** COVID-19, SARS-CoV-2, environmental contamination

## Abstract

The contamination of patients’ surroundings by severe acute respiratory syndrome coronavirus 2 (SARS-CoV-2) remains understudied. We sampled the surroundings and the air of six negative-pressure non-intensive care unit (non-ICU) rooms in a designated isolation ward in Chengdu, China, that were occupied by 13 laboratory-confirmed coronavirus disease 2019 (COVID-19) patients who had returned from overseas travel, including 2 asymptomatic patients. A total of 44 of 112 (39.3%) surface samples were positive for SARS-CoV-2 as detected by real-time PCR, suggesting extensive contamination, although all of the air samples were negative. In particular, in a single room occupied by an asymptomatic patient, four sites were SARS-CoV-2 positive, highlighting that asymptomatic COVID-19 patients do contaminate their surroundings and impose risks for others with close contact. Placement of COVID-19 patients in rooms with negative pressure may bring a false feeling of safety, and the importance of rigorous environment cleaning should be emphasized.

**IMPORTANCE** Although it has been well recognized that the virus SARS-CoV-2, the causative agent of COVID-19, can be acquired by exposure to fomites, surprisingly, the contamination of patients’ surroundings by SARS-CoV-2 is largely unknown, as there have been few studies. We performed an environmental sampling study for 13 laboratory-confirmed COVID-19 patients and found extensive contamination of patients’ surroundings. In particular, we found that asymptomatic COVID-19 patients contaminated their surroundings and therefore imposed risks for other people. Environment cleaning should be emphasized in negative-pressure rooms. The findings may be useful to guide infection control practice to protect health care workers.

## Abstract (precis)

Although it has been well recognized that the virus SARS-CoV-2, the causative agent of COVID-19, can be acquired by exposure to fomites, surprisingly, the contamination of patients’ surroundings by SARS-CoV-2 is largely unknown, as there have been few studies. We performed an environmental sampling study for 13 laboratory-confirmed COVID-19 patients and found extensive contamination of patients’ surroundings. In particular, we found that asymptomatic COVID-19 patients contaminated their surroundings and therefore imposed risks for other people. Environment cleaning should be emphasized in negative-pressure rooms. The findings may be useful to guide infection control practice to protect health care workers.

## OBSERVATION

Coronavirus disease 2019 (COVID-19) caused by severe acute respiratory syndrome coronavirus 2 (SARS-CoV-2) (1) has emerged as a global pandemic (2) and the top public health priority for many countries. Hospitalized patients with COVID-19 may contaminate their surroundings and impose risks for health care workers and cleaners (3). However, data about the contamination of SARS-CoV-2 in patient rooms are scarce. We therefore performed an environment sampling study.

## 

### Patients and settings.

On April 2, we sampled six negative-pressure rooms (12 air exchanges per hour) with toilets in a dedicated non-intensive care unit (non-ICU) isolation ward in Chengdu, China. There were 13 laboratory-confirmed COVID-19 patients hospitalized in the six rooms, which included one single-bed, two three-bed, and three two-bed rooms. All 13 COVID-19 patients were Chinese and had returned from overseas travel (5 cases from the United Kingdom, 3 from the United States, 2 from France, 1 from Iran, 1 from Thailand, and 1 from Malaysia; Table 1). On the day of environment sampling, the patients had been ill for 5 to 24 days and 11 patients had mild diseases according to the severity category of the national guidance on COVID-19 (4), while the remaining two (A and D) never developed symptoms (Table 1).

**TABLE 1:** Patient illness and results from clinical samples

Room	Patient	Country returned from	Result on the day of environment sampling		
			Disease severity	No. of days of illness	SARS-CoV-2-positive clinical sample category^*a*^
1	A	United Kingdom	Asymptomatic	18	Nasopharyngeal swab

2	B	Iran	Mild	24	Stool
	C	France	Mild	14	Nasopharyngeal swab
	D	Thailand	Asymptomatic	5	NA^*b*^

3	E	Malaysia	Mild	7	NA^*c*^
	F	United States	Mild	6	NA^*d*^

4	G	France	Mild	12	Nasopharyngeal swab
	H	United Kingdom	Mild	14	Nasopharyngeal swab

5	I	United States	Mild	10	—^*e*^
	J	United Kingdom	Mild	12	Nasopharyngeal swab
	K	United Kingdom	Mild	14	Nasopharyngeal swab

6	L	United States	Mild	9	Nasopharyngeal swab
	M	United Kingdom	Mild	10	Nasopharyngeal swab

a NA, not collected.

b A nasopharyngeal swab collected 2 days after the day of environmental sampling was positive for SARS-CoV-2.

c A nasopharyngeal swab collected 3 days before the day of environmental sampling was positive for SARS-CoV-2, and the patient was discharged the day after environmental sampling.

d A nasopharyngeal swab collected the day after environmental sampling was positive for SARS-CoV-2.

e —, the sample that was collected on the day of environmental sampling was negative, although a nasopharyngeal swab collected 3 days earlier had been positive for SARS-CoV-2; the patient was discharged the day after environmental sampling.

Environment sampling was scheduled on the day when nasopharyngeal swabs were collected from 10 patients, and stool was collected from 3 of the 10 as part of the routine care. Nine patients had a SARS-CoV-2-positive sample, consisting of either a nasopharyngeal swab sample (*n* = 8) or a stool sample (*n* = 1), as tested by real-time reverse transcriptase-PCR (RT-PCR; see below). Of the remaining three patients, one was discharged in the next day and a SARS-CoV-2-positive nasopharyngeal swab was collected from the other two in the following 1 or 2 days. This environment sampling was approved by the Ethics Committee of West China Hospital with oral informed consent having been obtained.

### Environmental surface sampling.

The rooms and toilets were cleaned and disinfected by nurses twice daily using a 2,000-mg/liter chlorine solution. We sampled frequently touched surfaces and the floor at 14 sites in patient rooms within 4 to 7 h after the first daily cleaning. The sites included the entire surface of bedrails, room and toilet door handles, light switches, foot flush buttons, sink rims, sink and toilet bowls and drains, and 1,200-cm^2^ (30-cm-by-40-cm) surfaces of bedside tables, bedsheets, pillows, equipment belts on walls, floors, and air exhaust outlets (Table 2). Each site was sampled using sterile swabs (Copan; Brescia, Italy) premoistened with a viral transportation solution (Longsee; Guangzhou, China).

**TABLE 2:** Environment sampling results

Site	No. of positive samples/total no. of samples (%)						
	Room 1, patient A	Room 2, patients B, C, and D	Room 3, patients E and F	Room 4, patients G and H	Room 5, patients I, J, and K	Room 6, patients L and M	Total
Patients’ rooms							
Door handle	0/1	1/1	1/1	0/1	0/1	0/1	2/6 (33.3)
Bedrail	1/1	3/3	1/2	1/2	0/3	1/2	7/13 (53.9)
Bedside table	0/1	3/3	1/2	0/2	0/2	0/1	4/11 (36.4)
Equipment belt on wall	0/2	2/2	2/2	0/2	0/2	0/1	4/11 (36.4)
Floor	0/1	1/1	0/1	0/1	0/1	1/1	2/6 (33.3)
Pillow	1/1	2/3	1/2	2/2	0/3	0/1	6/12 (50.0)
Bedsheet	1/1	3/3	1/2	1/2	0/3	0/1	6/12 (50.0)
Air exhaust outlet	1/1	1/1	0/1	0/1	0/1	1/1	3/6 (50.0)

Toilet area							
Door handle	0/1	1/1	0/1	0/1	0/1	0/1	1/6 (16.7)
Light switch	—^*a*^	1/1	1/1	0/1	0/1	0/1	2/5 (40.0)
Sink internal bowl and drain	0/1	1/1	1/1	0/1	0/1	0/1	2/6 (33.3)
Sink external rim	0/1	1/1	0/1	0/1	0/1	0/1	1/6 (16.7)
Toilet bowl and drain	0/1	1/1	0/1	0/1	0/1	1/1	2/6 (33.3)
Foot flush button	0/1	1/1	0/1	0/1	1/1	0/1	2/6 (33.3)

Total	4/14 (28.6)	22/23 (95.7)	9/19 (47.4)	4/19 (20.1)	1/22 (4.6)	4/15 (26.7)	44/112 (39.3)

a —, not sampled.

### Air sampling.

We also sampled air between 10:30 a.m. and 13:00 p.m. during the routine medical activities using an air sampler (FSC-1V; Hongrui, Suzhou, China) with 0.22-μm-pore-size filter membranes for 15 min at 100 liters/min. The air sampler was placed about 0.6 m away from each patient and 1 m above the floor in each room. The filter membranes were wiped by the use of premoistened sterile swabs (Copan).

### RT-PCR for detecting SARS-CoV-2.

RT-PCR (Sansure Biotech; Changsha, China) targeting open reading frame 1a or 1b (ORF1ab) and the nucleocapsid protein (N) gene was used to detect SARS-CoV-2 (5).

A total of 112 surface samples were collected, and 44 (39.3%, 44/112) were positive for SARS-CoV-2 (Table 2). The SARS-CoV-2-positive rate ranged from 4.6% (in a triple room) to 95.7% (in another triple room) for individual rooms. The SARS-CoV-2-positive rate also ranged from 16.7% to 53.9% for the individual sites. Bedrails (53.9%), pillows (50.0%), bedsheets (50.0%), air exhaust outlets (50.0%), and light switches (40.0%) were the top five contaminated sites.

Air was sampled for all of the six patient rooms, but all of the air samples were negative for SARS-CoV-2. However, 3 (50%, 3/6) of 6 samples from air exhaust outlets in three rooms were positive for SARS-CoV-2 (Table 2). It appears that the patient surroundings in rooms with a SARS-CoV-2-positive air exhaust outlet are usually extensive contaminated (26.7% to 95.7%). It is possible that small virus-laden particles may be displaced by airflows and deposited on patient surroundings as suggested previously (4).

The findings suggest that the patient surroundings in this non-ICU negative-pressure isolation ward for COVID-19 patients with mild disease symptoms or no symptoms were extensively contaminated by SARS-CoV-2. In particular, in a single room with an asymptomatic patient, four sites, including the bedrail, pillow, bedsheet, and air exhaust outlet, were SARS-CoV-2 positive. This highlights that asymptomatic COVID-19 patients can contaminate their surroundings and therefore that persons who have direct contact with them such as their family members and health care workers can be exposed to SARS-CoV-2. Isolation of asymptomatic COVID-19 patients at home imposes risks to their family members. Shelter hospitals may be a better option (6). The findings also highlight that the importance of environment cleaning should be emphasized. The remarkably wide-ranging SARS-CoV-2-positive rates for individual rooms may also reflect differing levels of stringency with respect to room cleaning and disinfection. All of the six rooms were maintained under negative-pressure conditions, which may have provided a false feeling of safety and may have led to laxity with respect to essential measures such as environment cleaning. Such a false feeling of safety is potentially harmful and should therefore be avoided.

We are aware of limitations in this study. Although we collected 1,500 liters of air for each air sample, that amount represents a low volume compared to the whole space of the room. We tested only for viral nucleic acid and did not perform viral culture to test viability. Despite the limitations, we believe that the findings reported here may help to guide prevention and control of COVID-19.

In conclusion, surroundings of COVID-19 patients can be extensively contaminated and asymptomatic COVID-19 patients do contaminate their surroundings and impose risks for others in contact with them.
